# Increased Rotavirus Prevalence in Diarrheal Outbreak Precipitated by Localized Flooding, Solomon Islands, 2014

**DOI:** 10.3201/eid2205.151743

**Published:** 2016-05

**Authors:** Forrest K. Jones, Albert I. Ko, Chris Becha, Cynthia Joshua, Jennie Musto, Sarah Thomas, Axelle Ronsse, Carl D. Kirkwood, Alison Sio, Audrey Aumua, Eric J. Nilles

**Affiliations:** Yale School of Public Health, New Haven, Connecticut, USA (F.K. Jones, A.I. Ko);; Fundação Oswaldo Cruz, Salvador, Brazil (A.I. Ko);; Solomon Islands Ministry of Health and Medical Services, Honiara, Solomon Islands (C. Becha, C. Joshua, A. Sio);; World Health Organization, Honiara (C. Joshua, J. Musto, A. Aumua);; World Health Organization, Suva, Fiji (J. Musto, A. Ronsse, E.J. Nilles);; Murdoch Childrens Research Institute, Royal Children's Hospital, Parkville, Victoria, Australia (S. Thomas, C.D. Kirkwood);; La Trobe University, Melbourne, Victoria, Australia. (C.D. Kirkwood)

**Keywords:** Disease outbreaks, epidemics, floods, disasters, weather, rotavirus, G9P[8], diarrhea, Solomon Islands, Guadalcanal, Honiara, Pacific islands, Western Pacific Ocean, surveillance, climate change, enteric infections

## Abstract

Flooding on 1 of the Solomon Islands precipitated a nationwide epidemic of diarrhea that spread to regions unaffected by flooding and caused >6,000 cases and 27 deaths. Rotavirus was identified in 38% of case-patients tested in the city with the most flooding. Outbreak potential related to weather reinforces the need for global rotavirus vaccination.

Pacific Island nations are vulnerable to extreme weather events that are projected to increase in severity and frequency with global climate change and can be associated with substantial health impacts, including outbreaks of diarrheal illness and other diseases (*1*–*4*). During the first week of April 2014, a tropical depression caused extensive flooding in the city of Honiara (population 64,609, 2009 census) and the surrounding province of Guadalcanal (population 158,222). Honiara is the capital city of the island nation of the Solomon Islands (population 515,870), a nation which consists of 9 provinces and 992 islands in the Western Pacific (Figure 1, panel A). >10,000 residents into emergency evacuation shelters and causing 22 deaths by drowning and other injuries (*5*). On April 20, 2014, an outbreak of diarrhea was declared in Honiara (Figure 1, panel B). During the next 2 months, diarrhea outbreaks and diarrhea-related deaths were reported from multiple provinces across the country that were not affected by the flooding. We investigated and report the flood-related outbreak of diarrhea in Honiara and its subsequent nationwide spread. 

**Figure 1:**
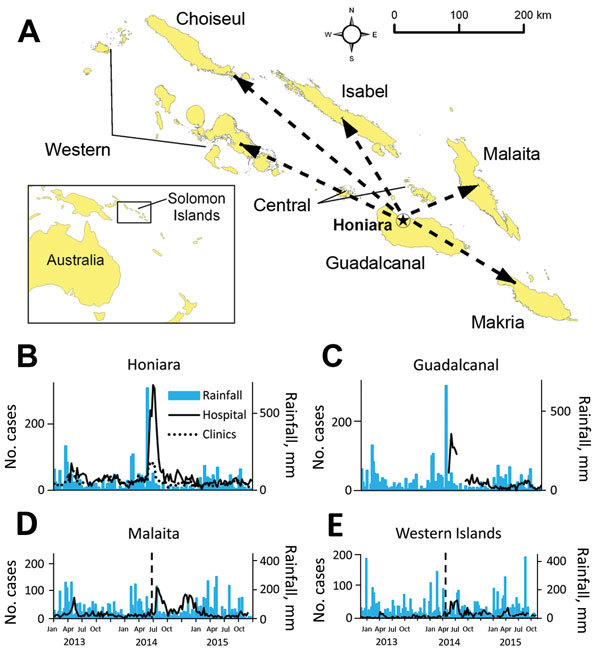
A) Spread of diarrheal disease in Solomon Islands after a postflooding outbreak in the capital city of Honiara, 2014, that resulted from a tropical depression. Dashed arrows indicate islands not affected by flooding where diarrheal outbreaks occurred. Two remote provinces, Temotu and Renell & Bellona, that did not report outbreaks are not included. B–E) Weekly rainfall measurements and outpatient diarrhea cases identified from the Pacific Syndromic Surveillance System (PSSS) database, December 31, 2012–August 30, 2015. The flooding occurred during April 3–5 in B) Honiara and C) other parts of Guadalcanal; vertical lines in panels D and E indicate the timing of the outbreak peak in Honiara and Guadalcanal. Cases of diarrhea are from PSSS weekly counts; Good Samaritan Hospital in Guadalcanal was designated as a PSSS site immediately postflood. Outpatient clinics in Honiara were Rove Clinic, Kukum Clinic, and Mataniko Clinic. Rainfall data presented for Guadalcanal Province is from Honiara.

## The Study

During March 31–April 6, 2014, in Honiara, a tropical depression caused 663 mm of rainfall, 10 times the mean weekly rainfall recorded during 2010–2013 (*6*). After the flooding event, use of the Pacific Syndromic Surveillance System (PSSS) (*7*), which collects weekly aggregated data on diarrhea and other syndromes from 4 health facilities in Honiara and 4 provincial hospitals, was transitioned to a postdisaster Early Warning Alert and Response Surveillance Network (EWARN) to enhance outbreak detection and response (*8*).

To characterize the outbreak, we identified nonbloody (>2 loose bowel movements in 24 hours) and bloody (any episode of loose bowel movement with visible blood) diarrhea from PSSS/EWARN databases and patient registries of 10 outpatient and inpatient facilities in Honiara and 6 provincial hospitals from the outbreak period, April 7–July 13, 2014. To verify that this was an outbreak and not normal seasonal fluctuation, we also reviewed longitudinal diarrhea data from PSSS sentinel sites during January 2013–August 2015.

The National Referral Hospital (NRH) in Honiara evaluated fecal samples from inpatients and outpatients who had diarrheal illness. Samples were routinely tested for *Salmonella enterica*, *Shigella* spp, *Vibrio cholera*, and rotavirus. A rapid diagnostic test (RDT), the SD BIOLINE Rota/Adeno Rapid kit (Standard Diagnostics, Inc., Yongin, South Korea), was used to test for rotavirus at the NRH (*9*). We genotyped rotavirus-positive samples as previously described (*10*) and calculated incidence rates using the Solomon Islands’ 2009 census. Negative binomial regressions and Fisher exact testing evaluated differences in rates and proportions testing positive.

We identified 4,087 diarrhea cases from the city of Honiara during the outbreak period, of which 3,664 cases (90%) were nonbloody. The mean number of weekly cases of diarrhea among PSSS sites in Honiara increased from 81.7 to 236.5 (rate ratio 2.90, 95% CI 2.13–3.96) from baseline to outbreak and baseline periods. (Table 1). The highest attack rate during the outbreak occurred in the <5 years age group (32%), which was >14× that observed for the ≥5 years age group (2%). During the outbreak, 6 of 9 provinces in the Solomon Islands reported diarrhea outbreaks, comprising the flood-affected province of Guadalcanal and 5 provinces that were not affected by the tropical depression and flooding (Malaita, Makira, Western, Isabel, and Choiseul) (Figure 1, panels C–E). Hospital-based surveillance identified 4,407 diarrhea cases, of which 1,626 (37%) were reported from provinces unaffected by flooding. During the outbreak, 27 diarrhea-related deaths were identified, including 10 from flood-affected provinces (3.6 deaths/1,000 case-patients) and 17 from provinces where no flooding occurred (8.6 deaths/1,000 case-patients). Of the 23 deaths with information on age, 21 (91%) were <5 years.

**Table 1:** Comparison of diarrhea prevalence before, during, and after outbreak linked to localized flooding, Solomon Islands, 2013–2015*

Facility†	Facility type	Location‡	No. cases (mean weekly cases)			Rate ratio (95% CI)
			2013§	2014¶	2015#	
National Referral Hospital	Hospital	Honiara	470 (36.2)	1969 (140.6)	347 (24.8)	4.65 (3.24–6.75)
Kukum	Clinic	Honiara	290 (20.7)	675 (48.2)	256 (18.3)	2.47 (1.67–3.70)
Rove	Clinic	Honiara	288 (20.6)	487 (34.8)	264 (18.9)	1.76 (1.18–2.66)
Good Samaritan Hospital	Hospital	Guadalcanal	NA	812 (116.0)	253 (18.1)	6.41 (4.43–9.42)
Kilu’ufi	Hospital	Malaita	248 (17.7)	627 (44.8)	112 (8.6)	3.36 (2.17–5.29)
Taro	Hospital	Choiseul	4 (0.3)	48 (3.4)	31 (2.2)	2.74 (1.18–6.71)
Gizo	Hospital	Western	72 (5.1)	427 (30.5)	140 (10.8)	3.88 (2.37–6.52)
*Source: Pacific Syndromic Surveillance System (PSSS) database. The rate ratio was calculated by comparing rates during the outbreak in 2014 and rates during years without outbreaks (2013 and 2015) and using a negative binomial regression. NA, not available. †One PSSS site in Honiara, Mataniko Clinic, is not shown because the clinic was partially destroyed by the flood and was not operational during the peak of the outbreak. ‡The city of Honiara is the capital of the Solomon Islands and is located in Guadalcanal Province; the remaining locations are provinces of the Solomon Islands. §April 8–July 14, 2013 (14 weeks). ¶April 7–July 13, 2014 (14 weeks). #April 6–July 12, 2015 (14 weeks).						

**Figure 2:**
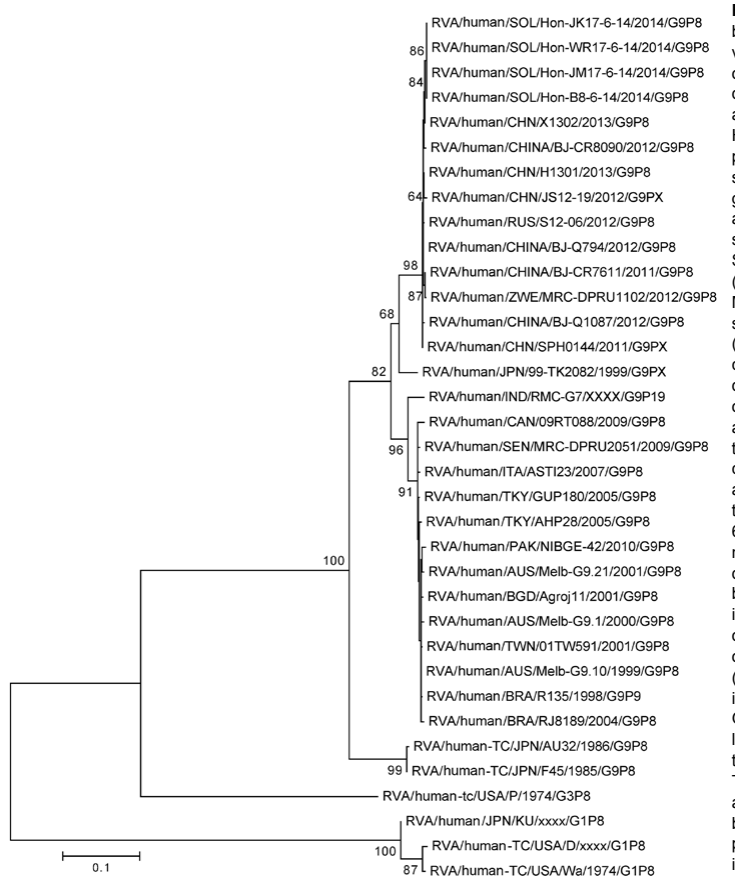
Nucleotide sequence–based phylogenetic tree of rotavirus viral protein (VP) 7 for isolates obtained in the Solomon Islands during an outbreak of diarrheal illness after flooding in the capital city of Honiara. Four isolates from different provinces had homologous VP7 sequences. We visually analyzed generated electropherograms and constructed contiguous DNA sequence files using the Sequencher Software program version 5.0.1 (Gene Codes Corp Inc., Ann Arbor, MI, USA). We performed nucleotide similarity searches using the BLAST (http://www.ncbi.nlm.nih.gov) and compared the nucleotide and deduced amino acid sequences of the VP7 gene with sequences available in GenBank possessing the entire open reading frame. We constructed multiple nucleotide and amino acid alignments using the MUSCLE algorithm in MEGA 6.0 (http://www.megasoftware.net/). Nucleotide and amino acid distance matrices were calculated by using the *p*-distance algorithm in MEGA 6.0. We selected the optimal evolutionary model based on the Akaike information criterion (corrected) ranking implemented in jModelTest (GitHub, Heidelberg, Germany) and generated maximum-likelihood phylogenetic trees using the nucleotide substitution model TrN+Gamma 4+I in MEGA 6.0, and assessed the robustness of branches by bootstrap analysis using 1,000 pseudoreplicate runs. Scale bar indicates base substitutions per site.

Among 61 fecal samples collected during the outbreak in Honiara, 23 (38%) tested positive in the rotavirus RDT, versus none of the 43 samples collected during the same period in 2013 (p<0.001) (Table 2); the proportion positive for other pathogens tested was similar for 2013 versus 2014. Of 5 samples collected in June during the outbreaks in non-flood–affected areas, 4 tested positive in the rotavirus RDT; each of the 4 positive samples were obtained from a different province (Choiseul, Isabel, Makira, and Malaita). Isolates from the 4 samples were identified as genotype G9P[8] and found to have identical VP4 and VP7 sequences (GenBank Accession nos. KU312099–KU312102). Samples were not available for genotyping from flood-affected areas. The VP7 isolates were most similar to strains circulating in China and Russia during 2011–2013 (Figure 2).

**Table 2:** Rotavirus rapid diagnostic tests of fecal samples before and during diarrheal outbreak precipitated by localized flooding, Honiara, Guadalcanal, Solomon Islands, 2013 and 2014*

Age group	% Positive (no. positive/no. tested)		p value§
	2013†	2014‡	
<5 y	0 (0/12)	41.9 (18/43)	0.005
≥5 y	0 (0/31)	23.5 (4/17)	0.012
Unknown	NA	100 (1/1)	NA
Total	0 (0/43)	37.7 (23/61)	<0.001

*Samples were collected and tested at the National Referral Hospital, Honiara; no rotavirus tests were conducted during the outbreak period in 2015. NA, not available. †April 8–July 14, 2013 (14 weeks). ‡April 7–July 13, 2014 (14 weeks). §By Fisher exact test.

## Conclusions

We report a nationwide epidemic of diarrhea in the Solomon Islands, which was precipitated by a focal extreme weather event. Disasters can facilitate transmission of infectious diseases through population displacement, overcrowding, limited access to clean water, and compromised hygiene and sanitation (*3*). An assessment to quantify the risk for disease outbreaks was done immediately after the floods and identified a high risk for diarrheal outbreaks in Honiara and Guadalcanal (E.J. Nilles, unpub. data). Using protocols developed after a tsunami emergency in 2013 (*11*) and insight gained by the experience, the Ministry of Health and Medical Services rapidly established an EWARN to strengthen disease detection and response capabilities. We report an increase in diarrheal cases shortly after the floods, and despite implementation of control measures, large outbreaks were detected in the flood-affected areas of Honiara and other parts of Guadalcanal. We subsequently identified outbreaks in multiple areas not affected by the flooding, indicating transmission from flood to non-flood affected areas.

Several factors suggest rotavirus was prevalent during the outbreak. The proportion of rotavirus-positive samples was 38% during 7 April–13 July, 2014, versus 0% during the same period in 2013, and increased to 55% during the peak of the outbreak from April 28–May 11. Outbreaks in non-flood affected provinces began soon after the peak of rotavirus transmission in Honiara. Illness and deaths caused by rotaviruses primarily affect the <5 years age group, unlike most other diarrheal agents with epidemic potential that affect all age groups (e.g., *Vibrio cholera, Shigella dysenteriae*, norovirus) (*12*). Finally, all 4 isolates from 4 non-affected provinces demonstrated 100% genetic homology of target genes, consistent with a common origin.

However, the small number of diarrheal samples tested for a small number of pathogens limited our ability to conclusively define the role of rotavirus versus other diarrheal pathogens. There was limited historical baseline data against which to compare our findings, but given the consistent collection of data through the PSSS during 2013–15, and the direct observations of multiple study authors (who were involved in outbreak response activities) of substantial surges in diarrhea cases throughout the country, we are confident that this was an outbreak and not seasonal fluctuation. We only included ambulatory cases and did not evaluate hospitalizations.

Improved understanding of the health implications of changing climate patterns is necessary to drive evidence-based mitigation strategies. Post-disaster early warning alert and response networks can ensure the rapid detection of, and response to, disease outbreaks that are likely to increase as climate change leads to more severe extreme weather events. Countries at risk for extreme weather events and other disasters, including many Pacific island nations, should ensure protocols and plans have been tested and are in place and to rapidly enhance disease detection and response capacities. Rotavirus may cause or contribute to epidemics in the post-disaster setting, emphasizing the importance of implementing global childhood rotavirus immunization guidelines (*13*).
