# Correction: Vol. 22, No. 5

**DOI:** 10.3201/eid2208.C22208

**Published:** 2016-08

**Keywords:** errata, erratum, correction

Misspellings were corrected and the term “prevalence” has been replaced by “case counts” in the Table 1 title in Increased Rotavirus Prevalence in Diarrheal Outbreak Precipitated by Localized Flooding, Solomon Islands, 2014 (F.K. Jones et al.). The article has been corrected online (http://wwwnc.cdc.gov/eid/article/22/5/15-1743_article).

